# Epitranscriptomics and cervical cancer: the emerging role of m^6^A, m^5^C and m^1^A RNA modifications

**DOI:** 10.1017/erm.2024.20

**Published:** 2024-10-08

**Authors:** Akshat D. Modi, Hira Zahid, Ashlyn Chase Southerland, Dharmeshkumar M. Modi

**Affiliations:** 1 Department of Biological Sciences, University of Toronto, Scarborough, Canada; 2 Department of Biology, University of Toronto, Mississauga, Canada; 3 Department of Health Sciences, California State University, Los Angeles, USA; 4 Department of Pharmacy, Silver Oak University, Ahmedabad, India

**Keywords:** anti-cancer therapies, cervical cancer, diagnostic biomarkers, epitranscriptomics, gene expression, m^1^A, m^5^C, m^6^A, RNA modifications, RNA-editing proteins

## Abstract

Cervical cancer (CC), one of the most prevalent and detrimental gynaecologic cancers, evolves through genetic and epigenetic alterations resulting in the promotion of oncogenic activity and dysfunction of tumour-suppressing mechanisms. Despite medical advancement, the prognosis for advanced-stage patients remains extremely low due to high recurrence rates and resistance to existing treatments. Thereby, the search for potential prognostic biomarkers is heightened to unravel new modalities of CC pathogenesis and to develop novel anti-cancer therapies. Epitranscriptomic modifications, reversible epigenetic RNA modifications, regulate various biological processes by deciding RNA fate to mediating RNA interactions. This narrative review provides insight into the cellular and molecular roles of endogenous RNA-editing proteins and their associated epitranscriptomic modifications, especially *N^6^*-methyladenosine (m^6^A), 5-methylcytosine (m^5^C) and *N^1^*-methyladenosine (m^1^A), in governing the development, progression and metastasis of CC. We discussed the in-depth epitranscriptomic mechanisms underlying the regulation of over 50 RNAs responsible for tumorigenesis, proliferation, migration, invasion, survival, autophagy, stemness, epithelial-mesenchymal transition, metabolism (glucose, lipid, glutamate and glutamine), resistance (drug and radiation), angiogenesis and recurrence of CC. Additionally, we provided a concise overview of the therapeutic potential of targeting the altered expression of endogenous RNA-editing proteins and aberrant deposition of RNA modifications on both coding and non-coding RNAs in CC.

## Introduction

Gynaecologic cancers encompass life-threatening malignancies that can affect the vulva, vagina, cervix and uterus, with the potential to spread to other organs associated with the functioning of the female reproductive system (Refs 1, 2, 3). In particular, carcinomas of the cervix, ovaries and endometrium are the most prevalent gynaecologic cancers, collectively representing 95% of all diagnosed cases (Ref. 4). Cervical cancer (CC), comprising highly prevalent squamous cell carcinoma and rare adenocarcinomas, is the leading cause of gynaecologic cancer-related deaths among women, primarily affecting those between the ages of 35 and 44 (Refs 4, 5). CC currently presents several challenges, including elevated incidence and recurrence rates, resistance to current treatments, poor prognosis at advanced stages (i.e., 5-year survival rate consistently lower than 50%) and high mortality rates (Refs 6, 7, 8). Therefore, there is an urgent need to deepen our understanding of the pathogenesis and progression of CC to overcome those limitations, which would unravel novel diagnostic markers and anti-cancer therapeutics.

Cervical cancer cells undergo both epigenetic and genetic changes that play a significant role in the disease progression, including the dysregulation of tumour-suppressing agents and oncogenes, from low-grade squamous intra-epithelial lesions to metastatic cancer (Ref. 9). The research literature encompasses a variety of approaches aimed at understanding the mechanisms and components of these changes. Researchers have explored the role of DNA methylation, non-coding RNA and histone modifications in understanding CC initiation, as well as the potential impact on tumour immunity within the complex microbial landscape (Refs 3, 10). Technological advancements in genomic sequencing, particularly in studying epigenetic modifications, are continuously revolutionizing our understanding of the human genome and its health implications (Refs 11, 12). Modern analysis techniques involve mapping the location and abundance of epigenetic modifications by combining antibody immunoprecipitation and chemical administration with next-generation sequencing (Refs 11, 12).

**Figure 1.**
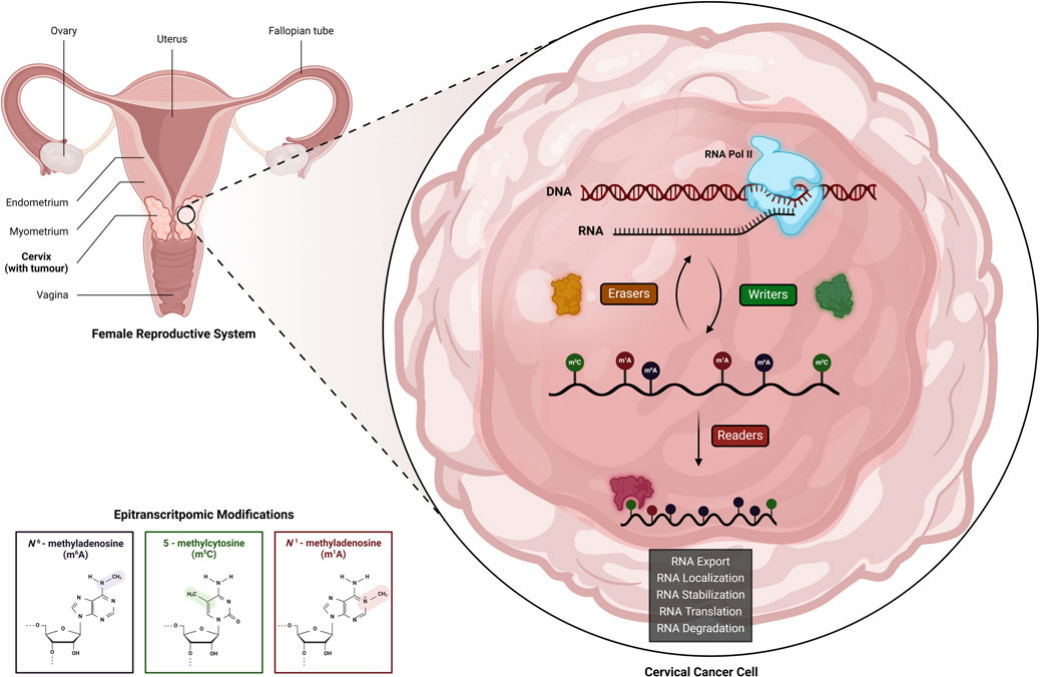
Epitranscriptomic mechanism in cervical cancer cells. DNA is transcribed into RNA, which undergoes various modifications including *N^6^*-methyladenosine (m^6^A), 5-methylcytosine (m^5^C) and *N^1^*-methyladenosine (m^1^A). These RNA modifications are regulated by specific endogenous RNA-editing proteins, categorized as: (1) ʻwriters', facilitating modification deposition; (2) ʻerasers', removing modifications; and (3) ʻreaders', interacting with modified RNA. This epitranscriptomic mechanism intricately governs RNA fate, influencing processes such as export, localization, stabilization, translation and degradation. Consequently, this modulation of genetic expression profoundly impacts cellular functions in cervical cancer. Created with BioRender.com.

Epitranscriptomic modifications, reversible epigenetic modifications of RNA, enable the regulation of various biological processes by RNA metabolism, localization, degradation, splicing, translation, stability, turnover and their intricate interactions. Groups of endogenous RNA-editing proteins have been identified to regulate epitranscriptomic modifications, including the ‘writers’ that facilitate the deposition of specific modifications, ‘erasers’ that remove particular modifications, and ‘readers’ that interpret the modifications and trigger downstream effects (Fig. 1) (Ref. 11). These modifications are observed across diverse RNA types, including messenger RNA (mRNA), transfer RNA (tRNA), ribosomal RNA (rRNA), enhancer RNA (eRNA), viral transfer RNA (vtRNA), small nuclear RNA (snRNA), non-coding small RNA (sncRNA), long non-coding RNA (lncRNA), microRNAs (miRNAs) and circular RNAs (circRNAs) (Refs 13, 14, 15). Sequencing technologies have revealed over 145 post-transcriptional RNA modifications, with RNA methylation comprising a significant portion, around 60%, of all RNA modifications (Refs 11, 12). The dynamic nature of RNA modifications enables swift cellular responses to environmental stimuli. The crucial role of RNA modifications in the fate of cancer tumour cells becomes apparent in their adaptation to rapidly changing and harsh conditions, such as those induced by drugs or stress. Epitranscriptomic modifications play a crucial role in the spatial and temporal expression of genes, and there is compelling evidence suggesting their involvement in tumour development, regulation and progression (Refs 9, 14). Epitranscriptomic modifications are linked to various hallmarks of cancer including survival, growth, restoration, differentiation, stress adaptation, invasion and drug resistance (Refs 16, 17, 18). Therefore, gaining a comprehensive understanding of molecular mechanisms, including the dysregulated endogenous RNA-editing proteins and epitranscriptomic modifications, that underlie the development and metastasis of CC is crucial for discovering diagnostic biomarkers, advancing therapeutic strategies and drug development. Notably, *N^6^*-methyladenosine (m^6^A), 5-methylcytosine (m^5^C) and *N^1^*-methyladenosine (m^1^A) are among the only epitranscriptomic modifications currently being researched in the context of CC.

In this comprehensive review, we have elucidated the intricate cellular and molecular mechanisms governed by endogenous RNA-editing proteins and their associated epitranscriptomic modifications, with a particular focus on especially m^6^A, m^5^C and m^1^A, in modulating expression of both coding and non-coding RNAs (i.e., oncogenes and oncosuppressor genes) within CC cells. This review delves into the multifaceted roles of epitranscriptome in regulating key features of CC. We highlight the pivotal implications of altered epitranscriptome in conferring resistance to conventional therapies and recurrence in CC. Furthermore, we provide a concise overview of the therapeutic avenues that emerge from targeting the altered expression of endogenous RNA-editing proteins and aberrant deposition of RNA modifications, underscoring the potential for precision medicine strategies in combating CC. Table 1 summarizes the role of epitranscriptomics in cervical cancer as discussed in this review.

**Table 1.** Epitranscriptomic Regulation of Gene Expression by Endogenous RNA-Editing Proteins Impacting Key Hallmarks of Cervical Cancer.

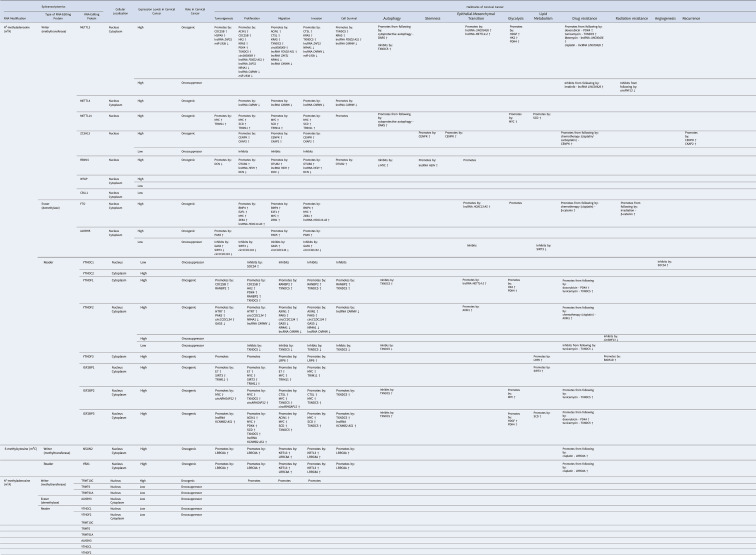

## Role of m^6^A modification in cervical cancer

**Figure 2.**
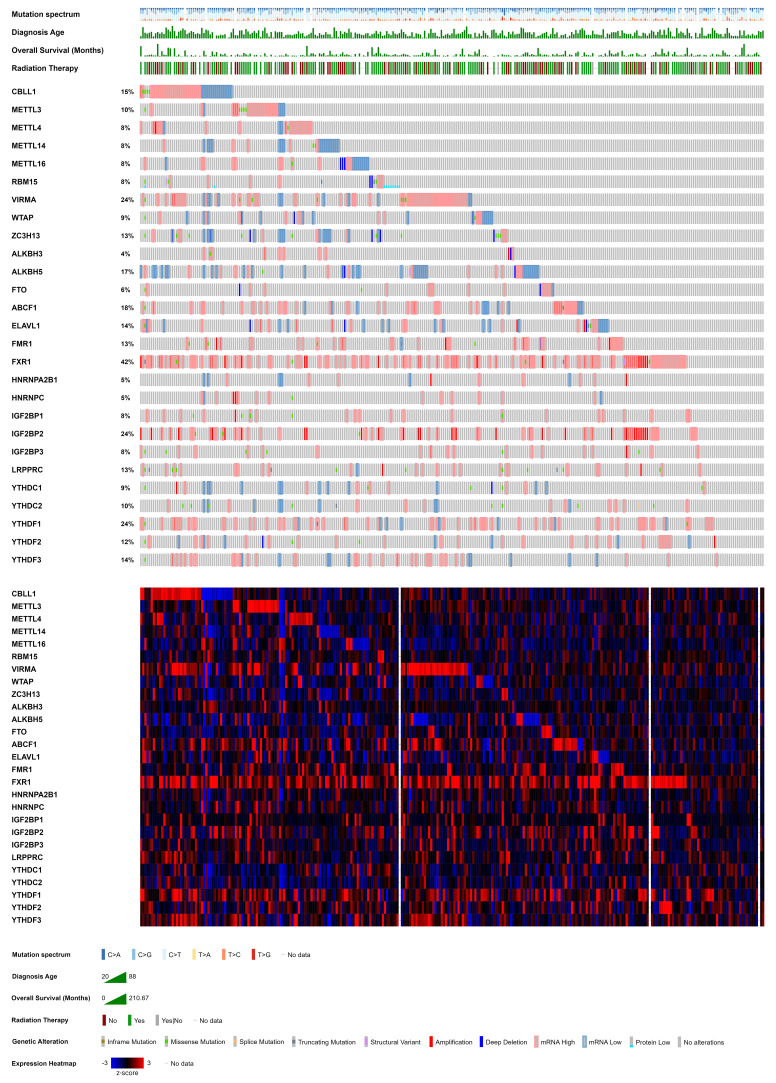
OncoPrint depicting the landscape of endogenous RNA-editing proteins responsible for *N^6^*-methyladenosine (m^6^A) modification in cervical cancer patients. Writers including CBLL1, METTL3/4/14/16, RBM15, VIRMA, WTAP and ZC3H13, as well as erasers ALKBH3/5 and FTO, are shown alongside readers such as ABCF1, ELAVL1, FMR1, FXR1, HNRNPA2B1, HNRNPC, IGF2BP1/2/3, LRPPRC, YTHDC1/2 and YTHDF1/2/3. Each column represents an individual patient sample and displays a comprehensive overview of the mutation spectrum, diagnosis age (years), overall survival (months), radiation therapy and genetic alterations, along with mRNA expression levels of m^6^A-associated endogenous RNA-editing proteins. mRNA expression is represented by *z*-scores relative to diploid samples (RNA Seq V2 RSEM). The Cancer Genome Atlas Program (TCGA) data of 297 cervical cancer patients were analysed and visualized using cBioPortal for Cancer Genomics (Refs 115, 116, 117).

*N^6^*-methyladenosine (m^6^A) RNA modification entails the methylation of the sixth nitrogen on the adenosine base and is currently the most researched chemical modification (Ref. 19). m^6^A modifications modulate RNA transcription, processing, splicing and translation to regulate oncogenic and tumour-suppressing gene activity (Ref. 19). CC cells have shown altered global m^6^A levels (Refs 20, 21), suggesting differential activity of writers and erasers promotes CC development and metastasis. The endogenous RNA-editing proteins for m^6^A modifications include (1) the writers METTL3/4/14/16, CBLL1, KIAA1429, ZC3H13, RBM15 and WTAP, (2) the erasers ALKBH3/5 and FTO, and (3) the readers YTHDC1/2, YTHDF1/2/3, HNRNPC, HNRNPA2B1, ELAVL1, ABCF1, FXR1, FMR1, LRPPRC and IGF2BP1/2/3. m^6^A regulators act as independent prognostic biomarkers, tumour microenvironment modulators and therapeutic targets for CC patients (Refs 22, 23, 24). Throughout the literature, various m^6^A-related independent prognostic signatures have been identified to predict CC patient survival including (1) ZC3H13, YTHDC1 and YTHDF1 (Ref. 25), (2) ZC3H13, RBMX, ALKBH5, YTHDC1/2 and YTHDF1 (Ref. 26), (3) METTL16, ZC3H13 and YTHDF1 (Ref. 27), (4) ZC3H13 and G3BP1 (Ref. 28), (4) ZC3H13, KIAA1429, HNRNPC and YTHDF1 (Ref. 13), and (5) IGF2BP1, IGF2BP2, HNRNPA2B1, YTHDF1, and RBM15 (Ref. 29). Moreover, ZC3H13 has shown the highest genetic alteration (especially deep deletion) frequency of 6% (Refs 25, 27), followed by 4% in LRPPRC and 3% in YTHDC2 (Refs 10, 27). ELAVL1, IGF2BP2, RBM15, WTAP, YTHDF2 and ZC3H13 show high frequencies of CNV deletions, while ABCF1, ALKBH3, FMR1, FXR1, IGF2BP2 and RBMX show high probabilities of CNV amplification (Refs 10, 28, 30). Among 297 cervical cancer patients, genetic alterations in endogenous RNA-editing proteins responsible for m^6^A modification were observed in 275 patients (93%) (Fig. 2), emphasizing the promising translational potential of these alterations as therapeutic targets and diagnostic markers warranting further investigation.

### m^6^A writers METTL3/4/14, ZC3H13, RBM15, WTAP and CBLL1

Methyltransferase 3 (METTL3), an oncogenic m^6^A writer, is highly expressed in CC and associated with poor prognosis in patients (Refs 20, 31, 32, 33). While METTL3 is highly expressed in tumours of all CC patients, human papillomavirus (HPV)-positive patients exhibit even higher METTL3 expression compared to HPV-negative patients (Ref. 34). Mechanistically, ETS proto-oncogene 1 (ETS1) activates the transcription of *METTL3* mRNA by mediating H3K4me3 and H3K27ac histone modifications through WDR5 and EP300, respectively, at the *METTL3* promoter region in CC cells (Ref. 33). Also, the binding of TATA-binding protein to the *METTL3* promoter region enhances METTL3 expression in CC cells (Refs 3, 35). High levels of METTL3 lead to alteration in mRNA stability, degradation and translation of several genes. This cascade of changes contributes to cell proliferation, migration, chemotaxis, lymph node metastasis, immunosuppressive tumour microenvironment (i.e., reduced immune cell infiltration) and survival of CC cells (Refs 3, 20, 32, 36, 37, 38). The involvement of METTL3 in cell cycle checkpoints and progression is critical for development and continuous growth of CC. The initiation of the G2/M phase is controlled by cell division cycle 25B (CDC25B), which stimulates the activation of CDK1/cyclin B and is considered an oncogene that is frequently altered in tumours (Ref. 36). High levels of METTL3 in the M phase upregulates CDC25B expression to promote cell cycle progression and tumorigenesis by inducing m^6^A modifications on *CDC25B* mRNA that are stabilized by m^6^A reader YTHDF1 (Ref. 36). Unique to METTL3, other key m^6^A writers do not exhibit remarkable expression during cell cycle progression (Ref. 36). Further interactions involve METTL3-induced m^6^A modifications on nuclear receptor *NR4A1* mRNA, interacting with m^6^A reader YTHDF2 and DDX6, promoting *NR4A1* mRNA degradation and facilitating malignancy in CC (Ref. 39). Overexpressing NR4A1 impairs CC progression by recruiting transcription repressing LSD1/HDAC1/CoREST complex that inhibits AKT1 expression and consequent activation of the Akt signalling pathway (Ref. 39). Moreover, the global increase in the transcription and translation rates within CC cells to support their malignant behaviour induces endoplasmic reticulum (ER) stress and demands proper protein folding to inhibit the activation of apoptosis pathways. TXNDC5, an ER protein that aids in correct protein folding, is highly expressed in CC patients (Ref. 33). METTL3 promotes CC cell proliferation and metastasis by inducing m^6^A modifications on the *TXNDC5* mRNA, which are stabilized and signalled for translation by m^6^A readers IGF2BP2/3 and YTHDF1, respectively (Ref. 33). Also, METTL3 downregulates YTHDF2 expression and prevents consequent YTHDF2-mediated degradation of *TXNDC5* mRNA (Ref. 33). METTL3 and TXNDC5 overexpression in CC reduces sensitivity to tunicamycin (i.e., glycosylation inhibitor) treatment, autophagy and apoptosis (i.e., low levels of Bax, active caspase 3 and LC3B-I/II) (Ref. 33). Furthermore, aggressiveness and metastasis of CC cells is mediated by METTL3-induced m^6^A modifications on apoptotic chromatin condensation inducer 1 (*ACIN1*) and cathepsin L (*CTSL*) mRNA, which are stabilized by IGF2BP3 and IGF2BP2, respectively, to upregulate their expression (Refs 32, 40). Overexpressing IGF2BP3 in the METTL3 knockdown cells can rescue the decreased ACIN1 levels by prolonging the half-life of its mRNA (Ref. 40). Overall, METTL3 knockdown inhibits cell proliferation (i.e., by arresting cancer cells at the G0/G1 phase of the cell cycle, increasing apoptosis, lowering *ACIN1* and *TXNDC5* mRNA stability), migration (i.e., by lowering *ACIN1* and *TXNDC5* mRNA stability) and invasion (Refs 20, 32, 40). The involvement of CD33+ myeloid-derived suppressor cells (MDSCs) add another layer to the complexity, with METTL3 expression positively associated with CD33+ MDSC density (Ref. 41). Given the role of MDSCs in enhancing tumour growth, establishing a pre-metastatic and immunosuppressive niche, and strengthening resistance to currently available immunotherapies for CC, high levels of both are correlated with shorter disease-free and overall survival of CC patients (Ref. 41). Hence, combining multiple therapies such as immune-checkpoint inhibition (i.e., anti-PD-1) and MDSC-targeted therapy with METTL3 inhibitor presents a promising therapeutic approach for CC patients. METTL3 plays a pivotal role in regulating the expression of factors packaged within tumour-derived exosomes crucial for intercellular communication within the tumour microenvironment (Ref. 42). It facilitates m^6^A modification on heat shock protein *HSPA9* mRNA, thereby enhancing both their stability and translation in CC (Ref. 42). This elevates the levels of exosomal mortalin HSPA9 protein, which correlates with tumour formation and progression (Ref. 42).

Methyltransferase 14 (METTL14), an oncogenic m^6^A writer, is highly expressed in both HPV-positive and HPV-negative patients and is associated with reduced overall survival (Refs 8, 43). Upregulated METTL14 promotes the proliferation, migration, invasion and survival of CC cells (Refs 8, 43). Conversely, METTL14 knockdown impairs the malignant properties, induces cell cycle arrest, inactivates the PI3 K/AKT/mTOR signalling pathway (i.e., reduces AKT and mTOR phosphorylation), upregulates pro-apoptotic protein expression (i.e., active Caspase 9, BAX and BIM) and downregulates anti-apoptotic protein expression (i.e., BCL-2) in CC cells (Ref. 8). METTL14 induces m^6^A modifications on tripartite motif-containing 11 (*TRIM11*) mRNA, a member of the E3 ubiquitin ligase family (Ref. 44). This modification enhances the stability of *TRIM11* mRNA through an interaction with IGF2BP1 (Ref. 44). Elevated levels of TRIM11 contribute to increased ubiquitination of PHLPP1, consequently activating the AKT signalling pathway, thereby promoting tumorigenesis, proliferation, migration and invasion of CC cells (Ref. 44). Suppressing TRIM11 expression to enhance PHLPP1 levels represents a promising therapeutic avenue for inhibiting tumour growth in CC (Ref. 44).

Zinc finger CCCH-type containing 13 (ZC3H13) is a highly expressed oncogenic m^6^A writer that modulates centromere protein K (CENPK) and cytoskeleton-associated protein 2 (CKAP2) expression to promote malignant properties, tumour stemness and chemoresistance in CC patients (Refs 45, 46). CENPK is a crucial protein in mitosis (especially chromosome segregation) while CKAP2, an intrinsically disordered protein, plays a key role in mitotic progression and exhibits cell-cycle-dependent expression (i.e., highest in the G2/M phase with localization in mitotic spindle and centrosome) (Refs 45, 46). ZC3H13-induced m^6^A modifications on *CENPK* and *CKAP2* mRNA upregulate their expression in CC, which is associated with cancer recurrence and shorter overall survival of patients (Refs 45, 46). The binding of CENPK and SOX6 disrupts the potential interaction of CENPK and *β*-catenin resulting in nuclear translocation and enhanced expression of *β*-catenin, p53 ubiquitination, activated Wnt/*β*-catenin signalling pathway and inactivated p53 pathway (Ref. 45). This alteration in cell activity results in proliferation (i.e., enhanced DNA replication), stemness (i.e., correlated with CD133, EPCAM, OCT4 and SOX2 expression), metastasis (i.e., enhanced epithelial-mesenchymal transition) and chemoresistance (i.e., enhanced DNA repair mechanism against cisplatin/carboplatin drugs) in CC (Ref. 45). CENPK knockdown impairs those malignant properties of CC (Ref. 45). While ZC3H13 inhibition reduces proliferation, migration and invasion of CC cells, overexpression of CKAP2 following ZC3H13 inhibition leads to partial restoration of those malignant properties (Refs 45, 46). This suggests that either inhibiting ZC3H13 or synergistically inhibiting both CENPK and CKAP2 presents a promising therapeutic approach for CC patients. Contradictory to the studies by Lin *et al*. (Ref. 45) and Zhang *et al*. (Ref. 46), Lu *et al*. (Ref. 23) showed down-regulation of ZC3H13 in CC cells and knockdown of ZC3H13 enhanced the proliferation, migration and invasion of CC cells; hence, requiring further investigation to fully elucidate the complexity of ZC3H13's role in CC.

RNA binding motif protein 15 (RBM15), an oncogenic m^6^A writer, is highly expressed in HPV-positive as compared to HPV-negative CC patients (Refs 34, 47, 48). The presence of HPV-E6 further exacerbates CC cell proliferation by enhancing intracellular *RBM15* mRNA accumulation (i.e., inhibits its degradation), RBM15-induced m^6^A modifications-mediated c-MYC upregulation and inhibition of autophagy (Ref. 34). Notably, HPV-E6 siRNA inhibits CC cell proliferation by promoting autophagy (Ref. 34). RBM15 promotes proliferation, metastasis and stemness of CC cells (Refs 49, 50). Moreover, RBM15-induced m^6^A modification on deubiquitinase otubain 2 (*OTUB2*) mRNA upregulates its expression, correlating with stage progression of CC and predicting poor prognosis (Ref. 48). Also, RBM15 downregulates decorin (DCN) expression by inducing m^6^A modification on *DCN* mRNA, thereby enhancing the progression of CC (Ref. 50). Conversely, RBM15 knockdown (i.e., upregulates DCN expression) supresses tumorigenesis, proliferation, migration and invasion of CC cells (Ref. 50). Silencing RBM15 has been shown to suppress the malignant properties of CC cells by inhibiting the JAK-STAT signalling pathway and reducing OTUB2 expression (Refs 47, 48, 50). Inhibition of OTUB2 promotes apoptosis and attenuates proliferation and metastasis of CC cells by downregulating the AKT/mTOR signalling pathway (Ref. 48). While a study conducted by Yuan *et al*. (2024) suggests that RBM15 might not a play role in apoptosis of CC cells (Ref. 49), indicating a need for further investigation into this aspect.

Methyltransferase 4 (METTL4) and WT1-associated protein (WTAP) are highly expressed m^6^A writers in CC cells, especially in HPV-positive patients (Refs 31, 34). The expression of WTAP in CC cells appears enigmatic, with conflicting reports indicating both upregulation (Ref. 34) and downregulation (Ref. 47), highlighting the complexity of its role and urging further investigation to reconcile these divergent observations. This difference in WTAP expression could be attributed to variations in CC cells samples, especially HPV status, but requires validation in future studies. Notably, the m^6^A writer Cbl proto-oncogene like 1 (CBLL1) exhibits significant downregulation in CC (Ref. 47), contrary to its overexpression observed in various other cancers. However, the oncogenic role of METTL4, WTAP and CBLL1 and underlying mechanisms remain largely unexplored.

### m^6^A erasers FTO and ALKBH5

Fat mass and obesity-associated protein (FTO), a prominent m^6^A eraser/demethylase, was initially identified for regulating body mass and obesity. However, emerging research has demonstrated its involvement in the proliferation of various cancers, including acute myeloid leukaemia, melanoma, and breast, lung, endometrial and pancreatic cancers (Refs 51, 52, 53, 54, 55, 56). In CC, FTO overexpression is associated with poor prognosis and regulates tumour cell proliferation, migration and invasion by upregulating the expression of cancer-promoting genes such as *E2F1*, *ZEB1* and *MYC* (Refs 19, 51, 57). FTO achieves this modulation by reducing the deposition of m^6^A modifications on their mRNA, thereby enhancing their translation efficiency (Refs 51, 57). Knocking down FTO impairs the expression of genes *E2F1* and *MYC*, leading to a reduction in cell proliferation, migration and invasion (Refs 51, 57). Notably, the ectopic expression of E2F1/ZEB1/MYC can restore the lost aggressiveness of CC (Refs 51, 57). Furthermore, FTO knockdown downregulates E2F1 downstream targets, impacts epithelial-mesenchymal transition and glycolysis while simultaneously activating the p53 pathway and DNA damage repair mechanisms (Ref. 51). FTO also modulates the m^6^A-deposition on genes involved with the BMP4/Hippo/YAP1/TAZ pathway, influencing CC proliferation, migration and invasion (Ref. 58). Importantly, BMP4 overexpression can restore the lost malignant behaviour in an FTO knockdown model (Ref. 58). Along with a crucial role in CC pathogenesis, FTO also interferes with the currently available treatments by enhancing the chemoradiotherapy resistance of CC cells (Ref. 55). FTO reduces the presence of m^6^A modifications on *β*-catenin mRNA, enhancing its translation efficiency, which upregulates the expression of the downstream DNA excision repair protein ERCC1 (Refs 3, 55, 59). Cells overexpressing FTO exhibit higher survival rates following cisplatin and irradiation treatment, while FTO inhibition increases the chemoradiotherapy sensitivity (Ref. 55). Inhibition of *β*-catenin counteracts FTO-induced chemoradiotherapy resistance in CC (Ref. 55). Elevated levels of FTO and *β*-catenin are associated with poorer prognosis of patients and reduced success rate of currently available cancer therapies (Ref. 55). Developing clinically safe drugs to inhibit oncogenic regulator FTO presents a promising therapeutic strategy for CC patients.

In contrast, m^6^A demethylase alkB homolog 5 (ALKBH5) acts as an oncosuppressor, limiting CC proliferation, migration, invasion and epithelial-mesenchymal transition (Ref. 60). Inhibition of ALKBH5 promotes the malignant behaviour of CC, downregulating E-cadherin expression and upregulating N-cadherin and vimentin expression (Ref. 60). Lower ALKBH5 levels are associated with a poorer prognosis in CC patients (Ref. 60). However, contradictory findings by Huo *et al*. (Ref. 61) suggest an oncogenic role of ALKBH5 in CC progression. The HPV E7 oncoprotein activates histone modifications (i.e., H3K4Me3 and H3K27Ac) via E2F1 and modulates post-translation modifications via DDX3, which promotes the expression of ALKBH5 in CC cells (Ref. 61). ALKBH5-mediated m^6^A demethylation on p21 activated kinase 5 (*PAK5*) mRNA stabilizes and enhances PAK5 expression in a YTHDF2-dependent manner, contributing to CC progression (Ref. 61). Also, METTL3, METTL14, FTO and ALKBH5 have been identified as regulators of the expression of the tumour suppressor DIRAS family GTPase1 (DIRAS1) (Ref. 62). While FTO and ALKBH5 play crucial roles in regulating malignant properties, non-coding RNAs and metabolism in CC (discussed in the below sections), the role and underlying mechanism of another m^6^A eraser, ALKBH3, remain unexplored.

### m^6^A readers YTHDC1/2, YTHDF1/2/3 and IGF2BP1/2/3

YTH *N^6^*-methyladenosine RNA binding protein C1 (YTHDC1), identified as a tumour-suppressing m^6^A reader, interacts with m^6^A modifications on the suppressor of cytokine signalling 4 (*SOCS4*) mRNA (Ref. 63). This interaction enhances SOCS4 expression, leading to the inhibition of angiogenesis and proliferation of CC cells (Ref. 63). Notably, CC patients exhibit low levels of YTHDC1 (Ref. 63). Overexpressing YTHDC1 counteracts CC progression by inhibiting proliferation, migration, invasion, impairing angiogenesis through reduced vascular endothelial growth factor A (VEGF) expression and facilitating CC cell apoptosis (Ref. 63). Conversely, YTH *N^6^*-methyladenosine RNA binding protein C2 (YTHDC2) is highly expressed in CC, yet its role and underlying mechanisms in pathogenesis remain elusive (Ref. 31).

YTH *N^6^*-methyladenosine RNA binding protein F1 (YTHDF1), an oncogenic m^6^A reader, exhibits high expression in CC, correlating with poor recurrence-free survival (Refs 20, 64). YTHDF1 upregulates RAN binding protein 2 (RANBP2) expression by interacting with m^6^A modifications on its mRNA (Ref. 64). This interaction promotes proliferation, migration and invasion while inhibiting apoptosis of CC cells (Ref. 64). YTHDF1 knockdown suppresses tumorigenesis and metastasis of CC cells and induces their apoptosis through downregulating RANBP2 expression (Ref. 64). While the RANBP2 knockdown impairs the migrative and invasive properties of YTHDF1-overexpressing cells (Ref. 64). Hence, targeting the YTHDF1-m^6^A-RANBP2 axis offers potential therapeutic avenues. YTH *N^6^*-methyladenosine RNA binding protein F2 (YTHDF2), an oncogenic m^6^A reader, interacts with the m^6^A modifications on the 5-hydroxytryptamine receptor 7 (*HTR7*) mRNA, contributing to tumorigenesis and dysregulated cell cycle in CC (Ref. 31). Elevated expression of YTHDF2 and its target, receptor HTR7, is associated with poor prognosis in CC patients (Refs 3, 31, 65). Inhibiting YTHDF2 emerges as a potential strategy to enhance the survival rate of CC patients. Additionally, YTHDF2 interacts with m^6^A modifications on the *AXIN1* mRNA, stabilizing its expression (Ref. 65). This interaction promotes CC progression and chemotherapy resistance of CC (Ref. 65). YTHDF2 inhibition reduces migration, invasion and epithelial-mesenchymal transition, and enhances cisplatin chemosensitivity through regulating AXIN1 expression and inhibiting the Wnt/*β*-catenin signalling pathway (Ref. 65). YTHDF2 knockdown arrests tumour cells in the S phase, impairing the growth of CC (Ref. 3). YTH *N^6^*-methyladenosine RNA binding protein F3 (YTHDF3), an oncogenic m^6^A reader, is upregulated in CC through transcriptional activation by the transcription factor SREBF1 (Ref. 66). YTHDF3 promotes the proliferation, migration and invasion of CC cells, thereby regulating tumorigenesis and lymph node metastasis (Ref. 66). Radiotherapy-resistant CC cells exhibit elevated expression of hepatocyte nuclear factor 1-alpha (HNF1*α*) (Ref. 67). Highly expressed HNF1*α* upregulates the expression of YTHDF3, which interacts with m^6^A modifications on DNA repair protein RAD51 homologue 4 (*RAD51D*) mRNA (Ref. 67). This interaction accelerates *RAD51D* mRNA translation, preventing and repairing radiation-induced DNA damage (i.e., breakage) to enhance cancer cell viability (Ref. 67). The HNF1*α*/YTHDF3/RAD51D axis is a critical regulatory mechanism in patients resistant to currently available radiotherapy. Targeting this pathway in conjunction with radiotherapy could promote the survival of advanced-stage CC patients.

Insulin-like growth factor 2 mRNA binding proteins 1/2/3 (IGF2BP1/2/3) are highly expressed oncogenic m^6^A readers that enhance the stability and translation efficiency of proto-oncogene MYC (Refs 68, 69, 70). PARKIN (i.e., E3 ubiquitin ligase) ubiquitinates IGF2BP3, promoting its degradation and loss of oncogenic function in normal cervical tissue (Ref. 69). However, low levels of PARKIN in CC cells result in IGF2BP3 overexpression, activating PI3 K and MAPK signalling pathways to promote tumorigenesis (Refs 69, 71). IGF2BPs knockdown reduces MYC expression, inhibiting proliferation, migration and invasion of CC cells (Refs 68, 70). HPV-induced carcinogenesis relies on the translation of viral early protein 7 (E7) in CC cells (Ref. 72). IGF2BP1 interacts with m^6^A modifications on the *E7* mRNA, stabilizing and promoting its translation (Ref. 72). Mild daily heat stress treatment destabilizes the oncotranscript complex, including IGF2BP1 and results in the formation of *E7*-IGF2BP1 aggregates (Ref. 72). These aggregates are targeted by the ubiquitin-proteasome system, downregulating E7 expression and reversing HPV-induced carcinogenesis (Ref. 72). This suggests an epitranscriptomic-associated heat-based treatment strategy for patients with HPV-positive CC.

Table 2 illustrates the synergistic and sequential interaction of writers/erasers and readers with RNA, elucidating their role in regulating the expression of over 50 oncogenes and oncosuppressors in cervical cancer. Consequently, the identification of therapeutic targets becomes imperative for disrupting this intricate network of endogenous RNA-editing proteins and advancing the development of effective therapies.

**Table 2.** Synergistic interaction of endogenous RNA-editing proteins to modulate epitranscriptomic modifications and expression of specific genes in cervical cancer

		RNA-editing proteins responsible for deposition or removal of RNA modifications							
		METTL3	METTL14	ZC3H13	RBM15	NSUN2	FTO	ALKBH5	Unknown/not required
RNA-editing proteins responsible for interacting with RNA modifications	YTHDC1								SOCS4 ↓
	YTHDF1	CDC25B ↑ HK2 ↑ PDK4 ↑ TXNDC5 ↑ lncRNA *METTL4-2* ↑							RANBP2 ↑
	YTHDF2	NR4A1 ↓ *circRNF13* ↓ lncRNA *CARMN* ↓	lncRNA *CARMN* ↓					PAK5 ↑ *circCCDC134* ↑ GAS5 ↓	AXIN1 ↑ HTR7 ↑
	YTHDF3								LRP6 ↑ RAD51D ↑
	IGF2BP1		TRIM11 ↑					SIRT3 ↑	E7 ↑ MYC ↑
	IGF2BP2	CTSL ↑ TXNDC5 ↑	MYC ↑						*circARHGAP12* ↑
	IGF2BP3	ACIN1 ↑ HDGF ↑ PDK4 ↑ TXNDC5 ↑	SCD ↑						MYC ↑ lncRNA *KCNMB2-AS1* ↑
	YBX1					KRT13 ↑ LRRC8A ↑			
	Unknown/not required	DARS ↑ HSPA9 ↑ KRAS ↑ *circ0000069* ↑ lncRNA *FOXD2-AS1* ↑ lncRNA *LINC00426* ↑ lncRNA *ZAFS1* *miR-193b* ↓	DARS ↑	CENPK ↑ CKAP2 ↑	c-MYC ↑ OTUB2 ↑ lncRNA *HEIH* ↑ DCN ↓		BMP4 ↑ E2F1 ↑ MYC ↑ ZEB1 ↑ β-catenin ↑ lncRNA *HOXC13-AS* ↑		

### m^6^A-associated long non-coding, micro, circular and PIWI-interacting RNAs

Long non-coding RNAs (lncRNAs), the largest group of non-coding RNA in mammals, manage around 70% of gene expression through DNA/RNA/protein interactions and have a potential role in cancer development (Refs 73, 74, 75). In CC, the oncogenic lncRNA *DARS-AS1* regulates cytoprotective autophagy in the hypoxic tumour microenvironment (Ref. 76). Hypoxia-inducible factor 1-alpha (HIF1*α*) transcriptionally upregulates the expression of *DARS-AS1* in CC cells (Ref. 76). *DARS-AS1* binds to the *DARS* mRNA to enhance its stability and recruits METLL3 and METTL14 to promote the translation of the *DARS* mRNA in CC cells (Ref. 76). Upregulated DARS modulates the expression of downstream targets, ATG3 and ATG5, to promote cryoprotective autophagy in CC (Ref. 76). This unveils the HIF1*α*/*DARS-AS1*/DARS/ATG5/ATG3 axis as a promising therapeutic target for CC patients. Another CC-associated lncRNA, *FOXD2-AS1*, is associated with poor prognosis in patients and promotes cell proliferation and migration in CC (Ref. 77). The expression of *FOXD2-AS1* is maintained by METTL3, which enhances its transcript stability through inducing m^6^A modifications (Ref. 77). *FOXD2-AS1* can lower *p21* mRNA expression by recruiting and supporting lysine-specific demethylase 1 (LSD1) (Ref. 77). *FOXD2-AS1* knockdown inhibits proliferative and migrative abilities, while promoting apoptosis in CC cells (Ref. 77). METTL3 also regulates lncRNA *METTL4-2*, promoting its expression through YTHDF1-mediated mechanisms, ultimately enhancing epithelial-mesenchymal transformation in CC (Ref. 78). METTL3 knockdown results in the upregulation of E-cadherin and downregulation of FN1, N-cadherin and vimentin (Ref. 78). The expression of lncRNA *HOXC13-AS* is upregulated and stabilized by the demethylase activity of FTO in CC cells (Ref. 79). *HOXC13-AS* upregulates frizzled class receptor 6 (FZD6) expression through H3K27ac modification induced by cAMP-response element binding protein (CBP) (Ref. 79). The FZD6-mediated activation of Wnt/*β*-catenin signalling pathway promotes cell proliferation and invasion and epithelial-mesenchymal transformation in CC (Ref. 79). Another m^6^A-regulaed lncRNA *LINC00426* plays a crucial role in promoting epithelial-mesenchymal transition in CC cells via *LINC00426*/*miR-200a-3p*/ZEB1 axis (Ref. 80). METTL3-induced m^6^A modification on *LINC00426* promotes its expression in CC cells, which makes those cells resistant to bleomycin and cisplatin and sensitive to imatinib (Ref. 80). LncRNA can also modulate the activity of RNA-editing proteins to promote epithelial-mesenchymal transition in CC (Ref. 81). LncRNA *LRRC75A-AS1* competitively binds with the IGF2BP1 protein, hindering its interaction with m^6^A modifications present on *SYVN1* mRNA (Ref. 81). This interference reduces the stability and translation of *SYVN1* mRNA, which inhibits the degradation of NLRP3 through SYVN1-mediated ubiquitination and activates IL-1*β*/Smad2/3 signalling pathways to facilitate the progression of epithelial-mesenchymal transition in CC (Ref. 81). Tumour-suppressing lncRNA *GAS5-AS1* is significantly downregulated in CC, leading to cell proliferation, migration and invasion, while its overexpression suppresses the development and metastasis of CC (Ref. 82). Reduced *GAS5-AS1* levels minimize the interactions between *GAS5* mRNA and ALKBH5 (i.e., regulates m^6^A modifications) (Ref. 82). YTHDF2 interacts with m^6^A modifications on *GAS5* mRNA, which destabilizes them and lowers the expression of GAS5 in CC (Ref. 82). While overexpression of *GAS5-AS1* upregulates tumour-suppressing GAS5 expression in the ALKBH5-m^6^A-YTHDF2-dependent pathway to inhibit CC tumorigenesis and metastasis (Ref. 82). m^6^A modification-associated regulation of lncRNA *MALAT1* expression has a critical role in CC (Ref. 83), however, its underlying upstream mechanism remains elusive. HPV-positive CC cells show high expression of *MALAT1* while silencing *MALAT1* attenuates the proliferative, migrative and invasive properties of those cells (Ref. 84). Also, silencing *MALAT1* modulates *miR-141-3p* expression, resulting in reduced ALKBH5 expression and consequent downregulation of MMP2 and MMP9, which suppresses migration and invasion of CC cells (Ref. 84). Moreover, the necroptosis-related lncRNA prognostic signature can predict the expression of m^6^A-associated writers, erasers and readers (Ref. 85). m^6^A-related lncRNAs can act as accurate biomarkers for predicting prognosis, tumour microenvironment, immune cell infiltration, response to immunotherapies and patient survival (Refs 73, 86, 87, 88, 89). Downregulated lncRNAs *AL109811.2*, *AC024270.4* and *AC008124.1* and upregulated lncRNAs *AC025176.1* and *RPP38-DT* are positively associated with the overall survival of CC patients, while the downregulated lncRNA *AC015922.2* and upregulated lncRNA *AC099850.4* are negatively associated with the overall survival of CC patients (Refs 73, 87).

Micro RNAs (miRNAs), a class of small non-coding RNAs, perform negative modulation of gene expression post-transcription and are widely known for their adamant roles in carcinogenesis (Ref. 90). Highly expressed lncRNA ZNFX1 antisense RNA 1 (*ZF-AS1*) in CC indicates poor survival of patients, higher metastatic potential and advanced FIGO stage (Ref. 91). Oncogenic *ZF-AS1* suppresses *miR-647* in a METTL3-mediated manner to promote CC development and metastasis while *ZF-AS1* knockdown inhibits cell proliferation, migration and invasion (Ref. 91). Overexpressing *miR-647* partially inhibits the malignant properties of CC (Ref. 91); hence, there would be missing parts to the METTL3-*ZF-AS1*-*miR-647* axis that needs to be explored. Highly expressed lncRNA *KCNMB2-AS1* in CC is associated with poor prognosis of patients while inhibiting *KCNMB2-AS1* suppresses proliferation and induces apoptosis of CC cells (Ref. 92). *KCNMB2-AS1* silences the expression of *miR-130b-5p* and *miR-4294* resulting in the upregulation of oncogenic IGF2BP3 (Ref. 92). IGF2BP3 interacts with the m^6^A modifications on *KCNMB2-AS1* to enhance its stability and expression (i.e., positive feedback loop), which results in pronounced tumorigenicity (Ref. 92). YTHDF2 interacts with METTL3/METTL14-induced m^6^A modification on tumour-suppressing lncRNA *CARMN*, promoting the degradation of *CARMN* (Ref. 93). *miR-21-5p* is a downstream target gene of *CARMN* that can bind to *CARMN* and negatively regulate expression (i.e., causes degradation) of *CARMN* (Ref. 93). Hence, targeting the interplay of m^6^A modification and *miR-21-5p* could reduce the occurrence and development of CC. RBM15 induces m^6^A modification to promote the stability and expression of lncRNA *HEIH*, which in turn promotes tumour cell proliferation, migration and stemness through the *miR-802*/EGFR axis (Ref. 49). METTL3-induced m^6^A modifications on tumour-suppressing *miR-193b* downregulate its expression in CC cells (Ref. 90). Low levels of *miR-193b* enable the overexpression of CCND1, which promotes deeper stromal invasion and tumorigenesis (Ref. 90). Overexpression of *miR-30c-5p* emerges as a promising therapeutic strategy to inhibit tumour growth and metastasis in CC (Ref. 94). *miR-30c-5p* exerts its effects by suppressing METTL3 expression, consequently reducing METTL3-induced m^6^A modifications on proto-oncogene *KRAS* mRNA. This leads to decreased expression of KRAS and promotes ferroptosis of CC cells (i.e., increases accumulation of Fe^2+^) (Ref. 94).

Circular RNAs (circRNAs) play a critical role in cancer progression by regulating gene expression, sequestering miRNA and RNA-binding proteins, and interfering with transcription and splicing mechanisms (Ref. 95). METTL3-induced m^6^A modifications increase the stability and expression of *circ0000069*, which suppresses *miR-4426* expression to promote CC proliferation and migration (Ref. 95). However, the downstream mechanism of *miR-4426* remains elusive. *hsa_circRNA_101996* acts as a *miR-8075* sponge and modulates the expression of microtubule nucleation factor TPX2 to inhibit cell proliferation, migration and invasion in CC (Ref. 59). Low levels of ALKBH5 in CC enable the presence of m^6^A modifications on *circCCDC134*, which significantly enhances its stability and expression in a YTHDF2-dependent manner (Ref. 96). *circCCDC134* regulates proto-oncogene MYB expression by recruiting p65 and functioning as a *miR-503-5p* sponge, which enhances *HIF1α* transcription and consequent CC development and metastasis (Ref. 96). Overexpression of ALKBH5 or HIF1*α* in CC cells prolongs or shortens the overall survival, respectively (Ref. 96). m^6^A-dependent upregulation of *circARHGAP12* in CC promotes tumorigenesis (Ref. 97). Moreover, *circARHGAP12* combines with *FOXM1* mRNA by interacting with IGF2BP2, which enhances *FOXM1* translation and consequent malignant behaviour of CC cells (Ref. 97). High expression levels of *circRNF13* promote the stability and expression of CXC motif chemokine ligand 1 (CXCL1), which results in enhanced radiotherapy resistance of CC cells (Ref. 98). Overexpressing METTL3 induces m^6^A modifications on *circRNF13* and promotes its YTHDF2-mediated degradation, which results in reduced expression of *circRNF13* and improved radiosensitivity in CC cells (i.e., similar to in CC cells with *circRNF13* inhibition) (Ref. 98).

Piwi-interacting RNAs (piRNAs) are widely expressed PIWI proteins-interacting small non-coding RNAs with dual roles in cancer, exhibiting both cancer-promoting and inhibiting properties (Refs 99, 100, 101). The highly expressed *piRNA-14633* in CC enhances the stability and expression of METTL14 in a concentration-dependent manner, leading to increased cytochrome CYP1B1 expression and promoting cell proliferation, migration and invasion (Ref. 99). Knockdown of *piRNA-14633* or METTL14 impairs the malignant properties of CC cells (Ref. 99). Additionally, the oncogenic role of highly expressed *piRNA-17458* CC involves the promotion of cell proliferation (i.e., S/G2 arrest), migration and invasion without influencing apoptosis (Ref. 102). *piRNA-17458* enhances the stability of *WTAP* mRNA (i.e., no effect on *METTL3*/*14*, *ALKBH5* and *FTO* mRNA stability), increasing m^6^A levels in CC cells and promote tumorigenesis (Ref. 102). Knockdown of *piRNA-17458* or WTAP abolishes the malignant properties of CC cells (Ref. 102).

### m^6^A-regulated metabolism

Understanding the impact of m^6^A modification on metabolism-related genes is crucial for unravelling the intricate mechanisms of cancer development and identifying potential therapeutic targets (Refs 20, 35). In CC, METTL3-induced m^6^A modifications on pyruvate dehydrogenase kinase 4 (*PDK4*) mRNA play a pivotal role in enhancing its stability (facilitated by IGF2BP3) and translation (facilitated by YTHDF1/eEF-2 complex) (Ref. 35). This cascade of events leads to the activation of glycolysis, characterized by increased glucose and oxygen consumption rates, and ATP generation pathways, ultimately promoting CC tumour growth (Ref. 35). ALKBH5 overexpression or METTL3 knockdown in CC cells demonstrates a decrease in glucose consumption, ATP levels, extracellular acidification rate and lactate production rate, while increasing the oxygen consumption rate (Refs 20, 35). Promoting the expression of glucose transporters and aerobic glycolysis enzymes becomes a strategy to increase glucose supply in the tumour microenvironment, heightening cell proliferation and inhibiting apoptosis (Refs 103, 104, 105). METTL3-induced m^6^A modifications on the growth factor *HDGF* mRNA enhance its stability and translation in an IGF2BP3-dependent manner (Ref. 20). This, in turn, promotes glycolysis by activating ENO2 and GLUT4 in CC cells (Ref. 20). METTL3-induced m^6^A modifications on hexokinase 2 (*HK2*) mRNA contribute to the enhancement of its stability and translation (mediated by YTHDF1) (Ref. 20). This process improves glycolytic capacity, highlighting the significance of METTL3 in driving the Warburg effect and aerobic glycolysis, ultimately promoting the proliferation of CC cells (Ref. 20). Exogenous expression of HPV oncogenes *E6*/*E7* enhances intracellular HK2 and GSK3*β* expression, contributing to CC tumorigenesis and metastasis (Ref. 106). Overexpressing FTO downregulates HK2 expression by inhibiting the nuclear export of *HK2* pre-mRNA, while GSK3*β* overexpression promotes ubiquitin-proteasomal FTO degradation (Ref. 106). E6/E7 proteins further regulate IGF2BP2 to interact with METTL14-induced m^6^A modifications on *MYC* mRNA, enhancing its translation to promote aerobic glycolysis, cancer development and metastasis (Ref. 103). The knockout of IGF2BP2 and E6/E7 demonstrates inhibitory effects on CC progression and glycolytic capacity (Ref. 103). METTL14 can boost glycolysis by activating the AMPK signalling pathway, leading to the production of lactic acid (Ref. 43). Elevated levels of lactic acid in the tumour microenvironment foster the M2 phenotype of macrophages, characterized by heightened expression of PD-1 (Ref. 43). This shift to the M2 phenotype correlates with reduced phagocytic activity, ultimately contributing to enhanced tumour growth (Ref. 43). The intricate involvement of ALKBH5 in lipid metabolism adds another layer to the intricate landscape of m^6^A-regulated metabolic pathways in CC. Low levels of tumour-suppressing ALKBH5 in CC are associated with enhanced fatty acid metabolism and poor patient prognosis (Ref. 60). Low levels of ALKBH5 enhance the presence of m^6^A modifications on *SIRT3* mRNA, which improves their stability and translation in an IGF2BP1-dependent manner (Ref. 60). Elevated expression of SIRT3 causes an increase in ACC1 expression resulting in enhanced lipid metabolism in CC cells (Ref. 60). Overexpressing ALKBH5 in CC cell lines results in removal of m^6^A modifications on *SIRT3* mRNA (i.e., lowers SIRT3 expression) and consequent reduction in *ACC1* expression, which suppresses lipid metabolism and malignant behaviour of CC cells (Ref. 60). IGF2BP3 interacts with METTL14-induced m6A modifications on stearoyl-CoA desaturase (*SCD*) mRNA, leading to upregulated SCD expression in CC cells (Ref. 70). Elevation in SCD levels accelerates lipid metabolism, ultimately promoting the proliferation and metastasis of CC cells (Ref. 70). YTHDF3 interacts with m^6^A modification on *LRP6* mRNA, boosting its translation efficiency in CC cells (Ref. 66). LRP6's pivotal role lies in activating the Wnt/ß-catenin signalling pathway, which in turn reprograms fatty acid metabolism to promote lymph node metastasis via the LRP6-YAP-VEGF-C axis in CC (Ref. 66). IGF2BP3 plays a critical role in enhancing glutamate and glutamine metabolism by stabilizing and upregulating the expression of *GLS* and *GLUD1* mRNA through an m^6^A-mediated mechanism (Ref. 71). This regulatory process leads to heightened lactate production and secretion, thereby facilitating Treg cell-mediated immune evasion (Ref. 71). The complex regulatory network involving m^6^A modifications, metabolic enzymes and oncogenic factors sheds light on the multifaceted nature of metabolic reprogramming in CC.

## Role of m^5^C modification in cervical cancer

**Figure 3.**
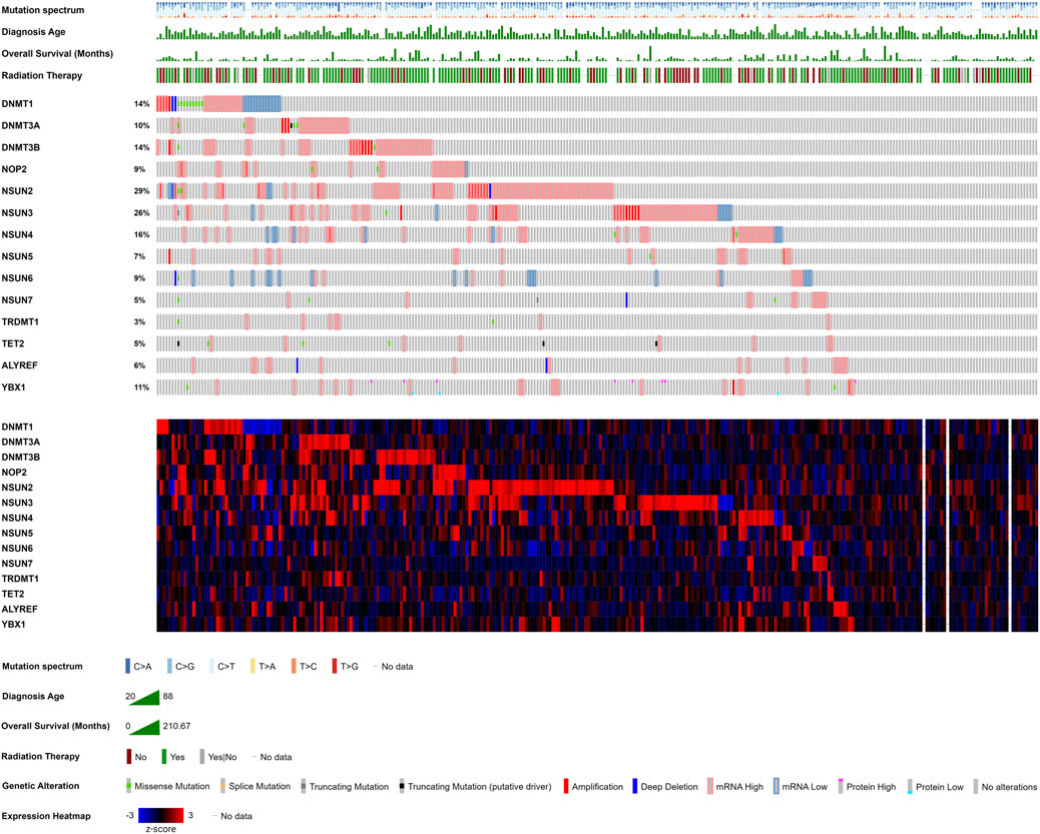
OncoPrint depicting the landscape of endogenous RNA-editing proteins responsible for 5-methylcytosine (m^5^C) modification in cervical cancer patients. Writers including DNMT1, DNMT3A/B, NOP2, NSUN2/3/4/5/6/7 and TRDMT1, as well as eraser TET2, are shown alongside readers such as ALYREF and YBX1. Each column represents an individual patient sample and displays a comprehensive overview of the mutation spectrum, diagnosis age (years), overall survival (months), radiation therapy and genetic alterations, along with mRNA expression levels of m^6^A-associated endogenous RNA-editing proteins. mRNA expression is represented by *z*-scores relative to diploid samples (RNA Seq V2 RSEM). The Cancer Genome Atlas Program (TCGA) data of 297 cervical cancer patients was analysed and visualized using cBioPortal for Cancer Genomics (Refs 115, 116, 117).

Recent research findings have shed light on the multifaceted role of 5-methylcytosine (m^5^C), a post-transcriptional modification characterized by cytosine methylation at the 5^th^ position, in various molecular processes. These encompass RNA export, fragmentation, translation, transcription, ribosome composition, tRNA homeostasis maintenance, stress regulation, codon-anticodon pairing, translation control, rRNA glioma sensitivity to stress-related enzyme NQO1 substrates, structural preservation of the tertiary rRNA–tRNA–mRNA complex, mRNA nuclear cytoplasmic-shuttling, splicing, DNA damage repair, migration, proliferation, development, differentiation, stability and stem cell augmentation (Ref. 15). Despite the well-established associations of m^5^C modifications with the development and aetiology of various cancers, autoimmune diseases and cardiovascular conditions, there exists a notable lack of research on their role and mechanisms in CC initiation and progression (Refs 14, 107, 108). This highlights the critical necessity to unravel the mechanisms and functionalities of m^5^C modifications in the specific context of CC. A comprehensive exploration of the functions of the writers, readers and erasers involved in the formation and removal of m^5^C modifications holds the promise of providing valuable insights into the intricate landscape of CC (Refs 14, 108). The writers or methyltransferases responsible for catalysing m^5^C modification include NSUN1/2/3/4/5/6/7, DNMT1, DNMT3A/B and TRDMT1 (Refs 1, 14, 109). On the other hand, TET2 acts as an eraser or demethylase, while ALYREF and YBX1 serve as readers or distinct effector proteins in the complex regulatory network of m^5^C modification (Refs 1, 14, 109). Among 297 cervical cancer patients, genetic alterations in endogenous RNA-editing proteins responsible for m^5^C modification were observed in 236 patients (79%) (Fig. 3), emphasizing the promising translational potential of these alterations as therapeutic targets and diagnostic markers warranting further investigation.

### m^5^C-associated prognostic gene signature

Genes intricately linked with m^5^C modification emerge as potent prognostic indicators in CC, offering accurate predictions of 1-, 3- and 5-year survival rates for patients (Ref. 14). Notably, a 4-gene signature comprising *CPE*, *FNDC3A*, *OPN3* and *VEGFA* has demonstrated remarkable prognostic capabilities (Ref. 14). Elevations in this gene signature within CC patients correlate with adverse prognoses, while therapeutic interventions targeting oncogenes *CPE*, *FNDC3A* or *VEGFA* exhibit promising outcomes by restraining cancer cell proliferation, migration and invasion (Ref. 14). The modulation of key m^5^C writers and erasers is intricately linked with the survival rates of CC patients. Downregulation of writers NSUN2/3/6, DNMT1 and DNMT3B and eraser TET2, coupled with the upregulation of writer NSUN5 and reader ALYREF, is associated with improved survival outcomes of CC patients (Ref. 14). However, the expression of writers NSUN1/4/7, TRDMT1 and DNMT3A appears to have no influence on patient survival rates (Ref. 14). Intriguingly, immune cell infiltration emerges as a pivotal factor influencing CC patient survival. Robust infiltration of activated CD8T cells, natural killer cells, macrophages and myeloid-derived suppressor cells is correlated with enhanced survival rates of CC patients (Ref. 14). Conversely, CC patients exhibiting central memory CD4 T cells and neutrophil infiltration tend to face a less favourable prognosis (Ref. 14). This nuanced understanding of gene signatures and m^5^C regulators opens avenues for a novel molecular diagnostic clinical test, facilitating prognostic risk assessment and identifying potential therapeutic targets for CC patients.

### m^5^C writer NSUN2 and reader YBX1

NSUN family of proteins emerges as pivotal players in tumour development and maintenance, offering potential m^5^C modified-oncogene biomarkers across various cancer types (Ref. 14). NSUN2, in particular, not only catalyses mRNA methylation but also contributes to critical cellular functions such as promoting cell proliferation, maintaining mitotic spindle stability, and responding to diverse cellular stressors (Refs 1, 110). In CC, the upregulation of NSUN2 takes centre stage, fostering the migration and invasion of cancer cells through m^5^C methylation on keratin 13 (*KRT13*) mRNA and consequent interaction/stabilization of those mRNA with highly expressed oncogenic reader YBX1 that promotes KRT13 expression (Refs 1, 111). KRT13, a 54-kDa type 1 acidic intermediate filament protein, is recognized as both tumour suppressor and tumour promoter depending on the type of cancer (Ref. 1). Potential therapeutic strategies involve inhibiting NSUN2 or introducing catalytically inactive mutations in NSUN2, disrupting the m^5^C-dependent NSUN2-YBX1-KRT13 axis to impede tumorigenesis in CC and improve patient survival. Inducing KRT13 overexpression can counteract the beneficial effects of inhibiting NSUN2 in CC, while overexpression of NSUN2 in KRT13 knockdown cells is unable to rescue the migration and invasion of CC (Ref. 1). While the impact of NSUN2 depletion on CC cell proliferation remains debatable, it consistently hampers their migration and invasion (Refs 1, 112). Notably, the impact of inhibiting YBX1 in CC pathogenesis remains unexplored, warranting further exploration.

LRRC8A (leucine-rich repeat-containing 8 volume-regulated anion channel subunit A), a regulator of cellular homeostasis and osmoregulation, assumes a dual role by promoting cell survival under physiological stresses and facilitating tumorigenesis in in vitro and in vivo models by suppressing apoptosis (Ref. 112). In CC, NSUN2 upregulation triggers m^5^C modification on *LRRC8A* mRNA, subsequently binding to the reader YBX1 and elevating mRNA stability, leading to enhanced LRRC8A expression (Ref. 112). This overexpression is associated with increased cell survival, growth, migration and invasion, thereby shortening recurrence-free survival for CC patients (Ref. 112). Knockdown of LRRC8A, conversely, inhibits CC cell proliferation, migration and invasion, accompanied by promoting the swelling and breaking of the cancer cells (Ref. 112). Additionally, LRRC8A knockdown reduces reactive oxygen species production and inactivates the PI3K/AKT signalling pathway, while inducing AKT activation in LRRC8A knockdown rescues the cell migration and inhibits Caspase-3 expression in CC (Ref. 112). Moreover, the LRRC8A knockdown cells are highly sensitive to cisplatin, suggesting its potential role in chemotherapy resistance in CC patients (Ref. 112). Consequently, targeting the NSUN2-mediated m^5^C-LRRC8A-YBX1 axis emerges as a promising therapeutic strategy to prevent the malignant properties of CC.

## Role of m^1^A modification in cervical cancer

**Figure 4.**
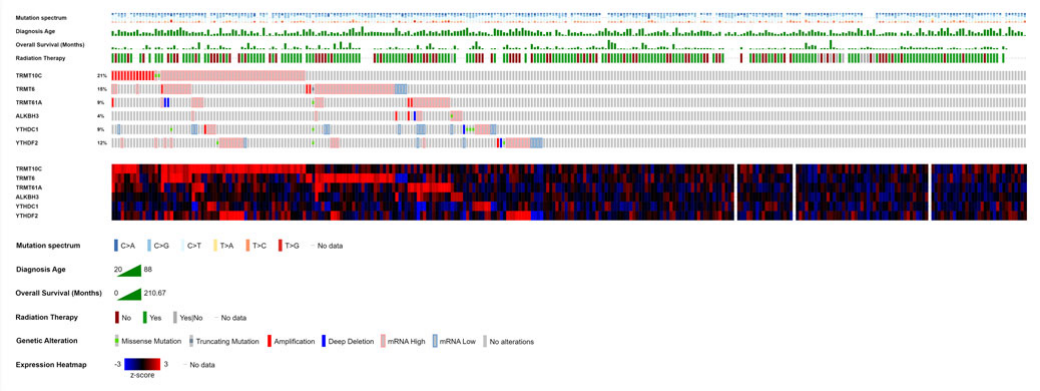
OncoPrint depicting the landscape of endogenous RNA-editing proteins responsible for *N^1^*-methyladenosine (m^1^A) modification in cervical cancer patients. Writers including TRMT10C, TRMT6 and TRMT61A, as well as eraser ALKBH3, are shown alongside readers such as YTHDC1 and YTHDF2. Each column represents an individual patient sample and displays a comprehensive overview of the mutation spectrum, diagnosis age (years), overall survival (months), radiation therapy and genetic alterations, along with mRNA expression levels of m^6^A-associated endogenous RNA-editing proteins. mRNA expression is represented by *z*-scores relative to diploid samples (RNA Seq V2 RSEM). The Cancer Genome Atlas Program (TCGA) data of 297 cervical cancer patients was analysed and visualized using cBioPortal for Cancer Genomics (Refs 115, 116, 117).

Existing literature highlights the significance of *N^1^*-methyladenosine (m^1^A), a post-transcriptional modification involving adenosine methylation at the *N^1^* position, in influencing RNA structure and protein interactions, with potential implications for gynaecological cancer cell proliferation (Refs 9, 113). Despite this, the specific role of m^1^A in CC remains largely underexplored. TRMT10C, an m^1^A writer, has garnered attention due to its distinct expression and functional consequences in these malignancies (Ref. 9). Elevated TRMT10C expression in CC has been associated with poor patient survival, and its silencing has demonstrated suppressive effects on cancer cell proliferation, migration and colony formation (Ref. 9). TRMT10C could potentially be associated with diverse cellular processes, including rRNA and tRNA metabolism, protein localization to the endoplasmic reticulum and chromosomes, nucleotide excision repair, endothelium and endoderm growth, integrin-mediated signalling and amoeboid-type cell migration (Ref. 9). Furthermore, advanced stages of CC are associated with a decreased expression of the m^1^A eraser ALKBH3 and m^1^A writer TRMT6 (Ref. 9). Conversely, high expressions of m^1^A writers TRMT6 and TRMT61A, along with m^1^A readers YTHDC1 and YTHDF2, have been correlated with better survival outcomes in CC patients, positioning them as promising prognostic biomarkers (Ref. 9). Notably, a significant correlation exists between the expression of m^1^A regulators and the expression of m^6^A and m^5^C regulators during oncogenesis (Refs 9, 114). Low-risk m^6^A/m^5^C/m^1^A-regulated genes (*CHAF1A*, *DUOX1*, *IGBP1* and *STAC3*) are associated with the infiltration of dendritic cells, macrophages, natural killer cells and T cells (Ref. 114). Conversely, high-risk m^6^A/m^5^C/m^1^A-regulated genes (*CA2*, *CUX1*, *IQGAP3*, *PTBP1*, *SLC2A1* and *STAC3*) are associated with infiltration of mast cells and poor survival duration of CC patients (Ref. 114). This intricate interplay between m^6^A/m^5^C/m^1^A regulatory genes showcases their association with the immune microenvironment and immunotherapy, suggesting that anti-CTLA-4 therapeutics and pazopanib might be most suitable for the high-risk group (Ref. 114). Among 297 cervical cancer patients, genetic alterations in endogenous RNA-editing proteins responsible for m^1^A modification were observed in 140 patients (47%) (Fig. 4), emphasizing the promising translational potential of these alterations as therapeutic targets and diagnostic markers warranting further investigation.

## Expert and topical summary

Epitranscriptomic modifications, reversible epigenetic RNA modifications, have emerged as a crucial factor in the development and progression of various cancers. This review explores the impact of epitranscriptomic modifications on CC, shedding light on endogenous RNA-editing proteins involved in this intricate process. Dysregulation of RNA modifications, specifically m^6^A, m^5^C and m^1^A, along with their associated writers, erasers and readers, significantly influences critical aspects of CC such as cell proliferation, migration, invasion, tumorigenicity and resistance to chemoradiotherapy. The review emphasizes the potential of targeting aberrant deposition of epitranscriptomic modifications by correcting the altered expression of associated RNA-editing proteins as a novel and promising therapeutic strategy for CC. The field of epitranscriptomics in CC is still in its infancy. With over 145 epitranscriptomic modifications and 20 of them being detectable with the currently available technologies, it presents a vast opportunity to explore the functional roles of unexplored RNA modifications in CC and opens avenues for developing drugs targeting epitranscriptomic modifications and RNA-editing proteins. In conclusion, epitranscriptomics stands out as a promising field in understanding the molecular mechanisms underlying CC. Further research should incorporate the use of single-cell RNA sequencing technology and multi-omics approach to elucidate the cell-specific functions of epitranscriptomic players and their cell-specific therapeutic potential in CC. The ongoing exploration and translation of those findings into clinically relevant diagnostic kits and treatment strategies holds a great promise that can potentially save lives and contribute to the well-being of women globally.
